# A Review on Serious Games for Dementia Care in Ageing Societies

**DOI:** 10.1109/JTEHM.2020.2998055

**Published:** 2020-05-28

**Authors:** Huansheng Ning, Rongyang Li, Xiaozhen Ye, Yudong Zhang, Lu Liu

**Affiliations:** 1 School of Computer and Communication EngineeringUniversity of Science and Technology Beijing 12507 Beijing100083China; 2 Beijing Engineering Research Center for Cyberspace Data Analysis and ApplicationsBeijing100083China; 3 School of InformaticsUniversity of Leicester 4488 LeicesterLE1 7RHU.K.

**Keywords:** Dementia care, serious games, ageing societies, therapy, assessment

## Abstract

Dementia is a severe disease threatening ageing societies, which not only causes great harm to patients both physically and psychologically but also places a heavy burden on patients’ families. Medications have been used for the treatment of dementia but with little success. However, serious games, as a new form of dementia therapy, stand out from various therapeutic methods and pave the way for dementia treatment. In the field of serious games for dementia care (SGDC) in ageing societies, there exists abundant research related to this topic. While, a detailed review of the development route and a category framework for characteristics of dementia are still needed. Besides, due to the large number of games, it is difficult to select out effective ones. Yet, there is no unified and comprehensive assessment methods for SGDC. So a reliable assessment model is worth studying. In this paper, we review these existing research work on SGDC from two perspectives: (1) the development of SGDC; (2) the different symptoms in different dementia stages. We also propose a comprehensive and professional assessment model of the therapeutic effectiveness of SGDC to compensate for the simplicity of existing assessment methods. Finally, a discussion related to SGDC is presented.

## Graphical abstract

We review the existing research on Serious Games for Dementia Care (SGDC) from two perspectives: (1) the development of SGDC; (2) the different symptoms in different dementia stages. We also propose and discuss a comprehensive and professional assessment model of the therapeutic effectiveness of SGDC to compensate for the simplicity of existing assessment methods.

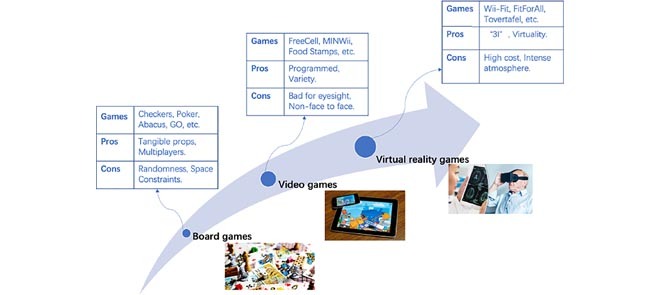

## I. Introduction

As a result of the decrease in birth-rates around the world, “aged society” and its related problems are frequent topics of research, among which dementia, as well as its treatment, is a major one. According to the World Health Organization (WHO), 91% of the total dementia patients are elderly people, making them the uppermost victims of this disease. Besides, there are 50 million people with dementia and the number of cases is increasing at a rate of 10 million per year. At first, research on the treatment of dementia concentrated mainly on drug therapies, but with little success [1]. Therefore, researchers have been trying to develop new methods to fight dementia. Serious games, with non-entertainment purposes (e.g. learning and treating) [2], stand out from a variety of methods. As is illustrated by Tárraga *et al.* [3], games therapy has a better effect on the cognitive abilities of patients compared with other therapies (e.g. traditional psychological stimulation and drug treatment), which showed that serious games have their place in the treatment of dementia.

Hence, serious games therapy has become a hotspot of dementia care research and a lot of related studies have been conducted. In the beginning, traditional board games, such as Huarongdao, Jigsaw, have been used as dementia care tools [4], [5]. With the development of technology, the use of some video games (e.g. Angry Birds, Kart Rider and so on) for dementia treatment is on the rise, which exhibits good performance [6]. However, what the above games all have in common is that they are not designed to treat dementia specifically. That is, these games do not have the explicit goal of treating dementia and are thus unable to offer targeted and appropriate treatment. In order to develop a more well-directed treatment for dementia, more and more customized games are developed. For example, games like Minwii, Big Brian Academy, Kitchen and Cooking are designed to treat cognitive impairment [7]–[9], and games like Wiifit, Wii Sports are designed to deal with movement disorder [10]. Although SGDC has been under study for a long time, there is no systematic review of the development process. To fill this gap, we have summarized the development stages of SGDC in hope of helping researchers to better understand its development within a short time.

After many long-term studies, numerous studies on SGDC have contributed to the SGDC field. Scholars reviewed these existing SGDC from different perspectives. For example, some researchers divided these serious games into non-electronic games and electronic games; Others reviewed them based on their different targeted symptoms (e.g. cognitive impairment, movement disorder, etc.) [1], [11]–[13]. However, many of them have a too broad scope to give researchers and developers a clear direction for further exploration. What’s more, different characteristics of dementia symptoms of different stages are also ignored. So, we analyze the different characteristics of symptoms in different dementia stages and present a category of SGDC based on it to offer reliable guidance for researchers.

Evidence have shown the effectiveness of serious games in dementia treatment, but reliable assessment methods are still needed for the game evaluation. Most of current researches realized such assessment via questionnaires and quizzes, such as Mini Mental State Examination (MMSE), Montreal Cognitive Assessment (MoCA) and so on [14], [15]. Some researchers used physiological signals (e.g. EEG, EDA, etc.) to explore the effects of games on people [16], [17]. Yet, these existing assessment methods are mainly based on one group of participants, which leads to random or uncertain results and lacks comprehensiveness and authority. To handle these problems, a model for assessing therapeutic effectiveness of SGDC is presented in this paper. This assessment model involves different groups (patients and professionals) and combines multiple methods (questionnaire tests, game results, professional reviews, physiological signals), which is different from other methods. This difference is also where our model is superior to others.

In this paper, we illustrate the development history of SGDC and review the existing research work according to the symptoms of different dementia stages systematically to offer explicit guidance for researchers. And then we propose an assessment model for SGDC to compensate for the simplicity of those existing assessment methods. The rest of the paper is organized as follows: Section 2 presents the development stages of SGDC, inculding the stage of board games, the stage of video games and the stage of virtual reality games; Next, Section 3 shows the category of SGDC based on the different symptoms of different dementia stages and the assessment model with multi-group participation and multi-method combination; Finally, the related discussion and prospect on this field are given in Section 4 and conclusion are drawn in Section 5.

## II. The Development of SGDC

**FIGURE 1.**
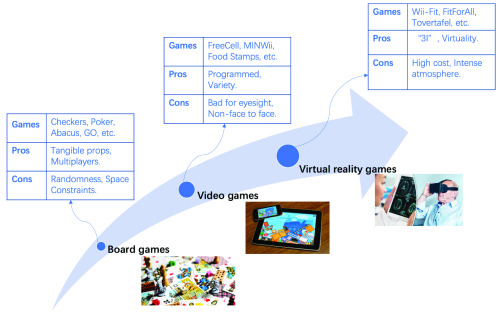
The evolution of SGDC.

Generally, people tend to turn to medication treatments for health issues, dementia is no exception. However, research has suggested that medication treatment for dementia has little effect whereas serious games are paving the way for dementia care [1], [3]. In this section, the evolution of SGDC is divided into 3 stages: board games, video games and virtual reality games. Fig. 1 shows the development of SGDC, including the different stages of SGDC, the representative games and its corresponding advantages and disadvantages. Additionally, we summarize related technologies and targeted symptoms of these three kinds of games in Table 1 to facilitate patients and caregivers to select out reasonable game types.

**TABLE 1:** Comparison Among Different Kinds of Games for SGDC

Game Type	Related Technologies	Targeted Symptoms
Board Games	—	mainly aimed at cognitive impairment
Video Games	Game Engine technologies, Computer Graphics, etc.	mainly aimed at cognitive impairment
VR Games	Somatosensory technology, 3D graphics generation technology, Sensor technology, etc.	cognitive impairment & physical disorder

### A. Board Games for SGDC

It is generally known that board games are a kind of multiplayer game which requires face-to-face interaction of players. In board games, players need to memorize things, communicate with each other, make reasonable inferences and so on, which can exercise one’s memory, improve language expression performance, cultivate social emotions and develop one’s reasoning abilities [18], [19]. Coincidentally, dementia impairs patients’ abilities of memory, cognition, emotional controls, which board games can help exercise. So, many researchers have applied board games to dementia care.

Experiments of Chen [20] verified that board games have a good effect on treating dementia. For example, checkers games can keep the brain cells active and develop one’s thinking ability; Abacus can train the fingers’ flexibility and the ability of hand-eye coordination and calculation; Poker is helpful to improve memory and thinking performance and exercise social communication abilities. In addition, Lin *et al.* [21] also proposed that Chinese GO can enhance patients’ social skills, reasoning ability and so on.

Generally speaking, board games are of benefit to patients with dementia. Moreover, the interactivity of such games makes participants communicate with each other. Therefore, it is easy to arouse patients’ interest of communicating with each other, which can develop their social skills under such game circumstances. Besides, tangible props are necessary for board games and participants have to interact with these props, which can strengthen touching sense and hands’ flexibility.

Board games also have their limitations. Due to the requirement of multiplayer participation, it is difficult to organize the play activities. For example, with space constraints, remote collaboration and control can not be achieved. Thus, carers and all patients must gather in one place. Apart from that, some board games, such as strategy games and role-playing games, are too difficult for dementia patients, so there are few board games left for dementia care which results in the simplicity of game forms. Additionally, the process of playing board games highly depends on players, which means the expected effect may not be achieved since the whole process can’t be programmed.

### B. Video Games for SGDC

Considering the development of computer technology and apparent limitations of board games in dementia care, electronic games (also called video games) have been considered as an alternative for dementia instead of board games. Video games are a kind of game that players interact with computers via electronic devices, such as mobile phones, pads, and laptops. Compared with board games, video games have a richer variety of types and some of them can be designed and developed according to the different symptoms of dementia. Consequently, video games have already caught many researchers’ attention in the SGDC field.

Typically, most board games can be designed as video games. Jimison *et al.* [22] combined Poker with a video game creating FreeCell card game. During the game, the data from the keyboard input and mouse movement is monitored to perform a cognitive assessment of elderly people with dementia.

In addition to those board game-based video games, there are many new video games designed in combination with other therapies. For instance, Benveniste *et al.* [7] applied music therapy into games and developed MINWii. In MINWii, players are guided to play a certain song or improvise on their own music on the virtual keyboard by caregivers. Results showed that their game can improve patients’ multi-sensory performance to alleviate symptoms. Chang *et al.* [23] combined the reminiscence therapy with their game, Food Stamps. In China, Food Stamps are the credential of buying food in the 1960s. To help patients restore their memories and cognitive ability, the game background was set in the 1960s and simulated the scene of buying food with Food Stamps to attract patients to play it.

Moreover, many researchers have integrated life skills into games and developed a series of games. Lopez-Martinez *et al.* [24] designed the game of gifts purchase. Gamers are asked to buy gifts for their family within a reasonable budget via the gaming platform on the computer. Such games not only can train the gamers’ planning and calculating abilities but also help them bond with their family. Manera *et al.* [25] applied Kitchen and Cooking game to the treatment of patients with mild cognitive impairment. “Kitchen and Cooking” is a game based on a cooking plot. During cooking, players need to select the correct ingredients and arrange the cooking process, which can exercise players’ planning and practical abilities. To train the ability of identifing items in life, Guia *et al.* [26] designed two interactive and collaborative games, namely Co-Brain Training Tool and AlzGame Tool, to help patients recognize items correctly, and in this way, enhance their cognitive performance.

Briefly, the application of video games in dementia care greatly promoted the development of SGDC. Video games not only can be substituted for board games but also have many new forms and categories of their own. Additionally, video games can better arouse players’ initiative, because of some interesting elements such as music, sound effects, visual effects, and quest rewards. Finally, the process of video games can be programmed, thus leading to a more desirable outcome.

Despite of these advantages mentioned above, video games do have their drawbacks. Firstly, due to the lack of face-to-face communication, communication abilities and social skills can not be fully exercised. Thus, during the game design process, developers should add more teamwork gaming tasks. Secondly, as such games are played on electronic devices, like pads, laptops, phones, players have to be in front of screens during the game, which is harmful to their eyesight. Therefore, gamers must rest their eyes timely and developers need to find new screen materials that can protect the eyes. Also, since elderly people tend to have poor eyesight, the text and icons in games may be hard to read on a screen for elderly people. Hence, when designing a game, designers should take into account this problem and try to use soft colors and brightness, set reasonable sizes for text, icons.

### C. Virtual Reality Games for SGDC

Virtual reality (VR) games, as the name suggests, are based on a virtual world to provide players with exciting visual, listening and touching experience as if they were in the real world [27], [28]. That is, games that can give players virtual and vivid feelings all belong to VR games, such as motion sensing games, and so on. As the continuous development of VR and somatosensory technology, serious games based on VR are of increasing popularity, and the use of VR games for dementia care is getting more attraction.

As the above description, motion sensing games are a kind of virtual games, which mainly focus on physical training for players. In this kind of game, players need to control the game process with their body movements. For example, in Wii-Fit [10], there are a set of alternative sports to choose for gamers, such as yoga, strength training, balance games and so on. Besides, in FitForAll [29], somatosensory devices and screens are used to provide a semi-virtual environment for gamers to do physical exercise. The above two games can exercise players’ balance and relieve motion impairment in addition to cognitive impairment. What’s more, some motion sensing games also can help with cognitive impairment. For instance, Urturi Breton *et al.* [30] designed KiMentia based on Kinect technology for elderly people with dementia. In KiMentia, gamers control the gaming process through their body movements to finish specific tasks, such as selecting out the correct syllables, choosing the correct letters or words. It’s showed that KiMentia has beneficial effects on patients’ mental and physical health. Additionally, He *et al.* [31] integrated gesture recognition with serious games. They use the 3D depth camera, instead of a regular camera, to achieve body gesture recognition and obtain game data. The game process contains memory practice, reasoning skills practice, calculation practice, and spatial recognition practice, for patients with dementia to gain a comprehensive rehabilitation training.

Moreover, there are some visual effects-focused VR games. For example, Tovertafel [32] applied the feature of interactivity. In tovertafel’s work, interactive projection technology is utilized to simulate the actual items, like flowers and leaves, which players can interact with during gaming. In this way, participants are more willing to play the game and do physical activities. Manera *et al.* [33] utilized Image-Based rendered VR to deal with cognitive impairment. They conducted a controlled experiment where participants are divided into two groups and need to find a specific goal in the given picture. The paper-based pictures are given to one group, and the VR-based pictures to the other. The result showed that the second group had more positive feedback, fewer errors and better performance during the game, which indicated that the virtual reality technology has great significance for SGDC.

In SGDC, virtual reality games have their own superiorities which are reflected by the 3I features of VR technology proposed by Burdea and Coiffet [34], namely immersion, interactivity and imagination. Immersion can arouse the enthusiasm of patients to join in the treatment which can enhance therapeutic effectiveness. Interactivity requires gamers to have interactions with game systems or other gamers which enables patients to gain comprehensive treatment. Imagination can inspire participants’ creativity and encourage their involvement which will stimulate their imagination and alleviate their impairments on cognitive, mental and physical levels. Apart from that, owing to the virtuality of VR games, even if some mistakes occur during the game, patients will not be hurt physically.

Nevertheless, there are still flaws in VR games for SGDC. With the high cost, some large-scale game devices based on VR technology are not affordable for most patients. Besides, the extreme sensory experience of VR games can also cause nausea. Additionally, most virtual reality games have a strong sense of immersion and stimulation to create a vivid game environment, thus, some elderly people with heart diseases can not play such a game. Consequently, when designing the game, developers should pay more attention to the control of the intensity of VR game so that not only can arouse the initiatives of gamers but also not give extreme stimulations which may have a bad impact on elderly people.

## III. SGDC for Different Dementia Stages and Its Assessment Model of Therapeutic Effectiveness

**FIGURE 2.**
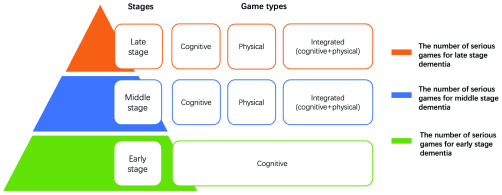
The category of SGDC based on different symptoms and stages of dementia.

**FIGURE 3.**
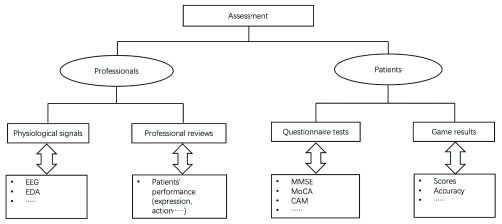
The assessment model of therapeutic effectiveness of SGDC.

In this section, the category based on different dementia stages and the assessment model of therapeutic effectiveness are given for SGDC. In Section A, we analyze the characteristics of different dementia stages. Based on it, we present a category for SGDC. Compared with other categories [1], [11]–[13], our category more focuses on the characteristics of different dementia stages, which can not only give researchers explicit guidance but also facilitate to bring patients a more well-directed treatment. Fig. 2 describes the category of SGDC. Table 2 summarizes the related serious games in different dementia stages. Additionally, in Section B, we propose an assessment model with multi-group participation and multi-method combination for the therapeutic effectiveness of SGDC. Our model involves different groups (patients and professionals) and combines multiple methods (questionnaire tests, game results, professional reviews, physiological signals), which is different from other methods. For clarity, Fig. 3 presents the assessment model of the therapeutic effectiveness of SGDC.

**TABLE 2:** Serious Games for Different Symptoms in Different Dementia Stages

Stages	Game types	Related literature	Games	Improved abilities
Early Stage	Cognitive	Byun et al. [35]	Fitt&Hick Game	Discernment Short-term memory Reaction
		Kitakoshi et al. [36]	Memory Game	Reaction Memory
		Boletsis et al. [37]	Pinball Recall	Problem solving Logical reasoning
		Anguera et al. [38]	NeuroRacer	Hand-eye coordination
		Tost et al. [39]	Smart Ageing	Memory Attention Spatial orientation
Middle Stage	Cognitive	Tong et al. [40]	Executive Timed Target Game	Reaction
		Imbeault et al. [41]	Point-click Game	Planning Objects recognizing
		Lin et al. [42]	Planting Game	Observation
		Hamada et al. [43]	Pet-type Robot	Communication Observation Body movement
		Tapus et al. [44], [45]	Socially Assistive Robot	Communication Memory
	Physical	Soares et al. [46]	SIRTET Game	Balance Muscle strength
		Jung et al. [47]	Wii	Overall well-being
		Agmon et al. [48]	WiiFit	Balance Attention and coordination Quick motor response
	Integrated (cognitive &physical)	Unbehaun et al. [49]	Exergame	Memory Limbs and muscles strength
		de-Urturi et al. [50]	Kinect-based Virtual Game	Body movement Objects recognizing
		Kayama et al. [51]	Kinect-based Exercise Game	Fall prevention Planning
		Chilukoti et al. [52]	Assistive Technology System	Muscle strength Problem solving Memory
		AAB Arntzen [53]	Learning Game	Cognitive abilities Physical abilities
Late Stage	Cognitive	N/A		
	Physical	N/A		
	Integrated (cognitive&physical)	N/A

### A. SGDC for the Different Stages of Dementia

Dementia is a dreadful disease, with in-depth research on the treatment of dementia, serious games played a significant role in dementia care. Only by understanding the different stages and symptoms of dementia can serious games provide patients with reasonable and effective treatment. According to WHO, the period of dementia is divided into 3 stages, the early stage, the middle stage, and the late stage. The early stage is the intermediate state between normal aging and dementia. In the early stage, patients will show symptoms, namely forgetfulness, being lost in familiar places, etc. These symptoms of early stage are collectively known as Mild Cognitive Impairment (MCI). In the middle stage, symptoms of the early stage worsen and new symptoms appear, including communication difficulties, poor self-care ability and so on which can be summarized as cognitive impairment and physical disorder. During the late stage, patients will be unaware of time and place, in addition to the inability of self-care, severe mental disorder. In order to offer patients a targeted treatment, we present the category of SGDC according to the above different stages and symptoms of dementia. Fig. 2 describes the category of SGDC based on different symptoms and stages of dementia.

#### 1) SGDC for Early-Stage Dementia

In the early stage, there will only be some cognitive changes, such as the mild decline in memory and thinking ability, which are hard to distinguish from normal aging. However, if the case can not be diagnosed and treated in time, it will step into the middle stage or late stage, causing greater harm to patients and their families. Therefore, a set of serious games are designed for cognitive screening and training.

The Fitt&Hick game and Memory game [35], [36] are both based on the observation of pictures. Their specific gaming tasks include identifying colors of pictures, learning the content or positions of pictures. The main purpose of them is to check if players suffer from cognitive impairments and train their various cognitive abilities, among them are rapid reactions, discernment skills, and long/short-term memory.

The above two games are simple types. There are also a list of slightly difficult games, such as rule-finding games, common skill games, life scene games and so on. Pinball Recall game [37], a rule-finding game, is developed by Costas Boletsis and Simon McCallum. In this game, an example is given to gamers as a hint for finding rules. After finding out the rule, gamers are required to finish the specific task accordingly. Apparently, their game can test and exercise players’ abilities of solving problems and logical reasoning. Apart from that, Anguera *et al.* [38] designed NeuroRacer, a common skill game, which simulates the driving scenario to let gamers drive on a highway with traffic signals in a virtual environment. During driving, gamers must comply with traffic regulations and control the car’s direction, speed, which can train players’ hand-eye-brain coordination and improve the level of cognition. Besides, Tost *et al.* [39] developed a 3D game, Smart Ageing, a life scene game, that simulates the daily life scene using 3D technology and guides the elderly to complete the specific living task to test or exercise their cognitive abilities, such as executive functions, divided attention, long/short-term memory, and spatial orientation and attention.

#### 2) SGDC for Middle-Stage Dementia

In the middle stage, not only cognitive impairment is getting more and more serious but also various disorders appear physically. So, the primary mission for this stage is treatment rather than detection. Therefore, many games are designed to treat cognitive and physical disorders according to the characteristics of symptoms of this stage.

To address symptoms of cognitive impairment, some games were proposed. Tong *et al.* [40] designed an Executive Timed Target Game which can enhance the reaction of patients. Also, Imbeault *et al.* [41] applied Point-and-click game based on the cooking scenario into the treatment of dementia. In the cooking scenario, gamers are required to finish a certain task, such as toasting bread, making coffee, to train patients’ abilities of planning and recognizing objects. Lin and Chen [42] designed a video game, Planting Game, which simulates a planting scenario. During the game, to ensure the healthy growth of trees, players need to adjust the parameters of luminance, temperature, etc. Results showed that the process of planting can improve the cognition of patients. Apart from these traditional video games, some serious games are designed based on robot therapy. Pet-type robot [43] and the Socially assistive robot [44], [45] guide players to interact with robots to exercise their various abilities such as observation, communication, memory, body movement and so on. Actually, since the shift from the early-stage dementia to its middle stage is a gradually varied process, there is no explicit boundary between the middle stage and the early stage for treating cognitive impairment in games’ application. So, it can be flexibly adjusted according to the specific situation of the patients.

Moreover, there are games designed to relieve physical disorders alone or address the cognitive impairments and relieve physical disorders simultaneously. For physical disorders, some studies have been done. For example, Soares *et al.* [46] applied the SIRTET game into the physical rehabilitation of the elderly. During the game, gamers need to hit the targets, avoid obstacles or hit and avoid objects at the same time which can train elder’s balance ability and muscle strength. Also, Wii or WiiFit have been used broadly in the treatment of physical disorders, Jung *et al.* [47] and Agmon *et al.* [48] conducted experiments to verify the effectiveness of Wii and WiiFit in the treatment respectively. Results showed that the above two games can improve patients’ balance, reaction, muscle strength and so on. In fact, since the physical activity is often accompanied by cognitive exercise. Hence, more games can address cognitive impairments and relive physical disorders at the same time. Most of which are somatosensory games. Unbehaun *et al.* [49], Urturi *et al.* [50], and Kayama *et al.* [51] developed different somatosensory games respectively. In their games, camera-based somatosensory technology is used to achieve the interaction between players and game systems. In the interactions, players have to finish various tasks, like sports, memorizing, plan making, which can enhance cognition (e.g. planning, memory, problem solving, etc.) and physical abilities (e.g. limbs and muscles strength, fall prevention, body movement, etc.) simultaneously. These all above three games used the somatosensory technology based on the camera. Also, some games utilized other sensors to capture player’s information. Chilukoti *et al.* [52] installed Hall effect sensor to a portable mini stationary bike to send players’ thoughts to the game system. In this game, players need to select out the correct option according to their judgment via pedaling the mini bike, which can improve their cognitive and physical abilities at the same time. In addition, AAB Arntzen [53] came up with the concepts and requirements of a serious game related to sport and dance activities which aimed to enhance cognitive and physical abilities of elderly people. Arntzen’s idea provided guidance for serious game design. For instance, designers should pay more attention to the interestingness, practicality (adapt to poor eyesight and hearing of the elderly), and effectiveness.

#### 3) SGDC for Late-Stage Dementia

According to WHO, late-stage patients are in bad condition. Their multiple abilities are significantly compromised, which results in patients having almost completely lost cognitive and motor functions. However, if serious games are applied as the treatment of dementia, people being treated are required to have the cognitive abilities to understand the gaming rules and have the physical strength to complete game tasks. Therefore, serious games are not suitable for the treatment of late-stage patients. Consequently, there are hardly any cases of existing serious games aimed at such patients.

Overall, in the early stage, the main purpose is to diagnose dementia and improve patients’ cognitive ability. Usually, common video games can meet the needs of this stage. While, in the middle stage, our priority is treatment rather than diagnosis. Furthermore, middle-stage games can not only exercise patients’ cognitive ability but also their physical ability, which makes somatosensory games more applicable to patients in the middle stage.

### B. The Assessment Model of Therapeutic Effectiveness

Serious games are widely used in the treatment of dementia. Accordingly, the effectiveness of serious games therapy must be guaranteed. Therefore, an effective assessment method for SGDC is necessary. Now there are many methods to evaluate the therapeutic effectiveness of games and all these methods are mainly done via questionnaire tests and physiological signals. Based on this, we further added two new modules into our assessment model, respectively game results and professional reviews. What’s more, the above four modules are based on different groups: questionnaire tests and game results are mainly based on patients’ feelings and performance, and the professional reviews and physiological signals are based on professional perspective [2]. In particular, our work aims at presenting a conceptual assessment model, which can provide researchers with a new road in the assessment of SGDC. Thus, the specific implementation of each module and the overall model requires further research and exploration. For clarity, Fig. 3 presents the assessment model of therapeutic effectiveness of SGDC.

Questionnaire tests are so far the most common methods to assess the therapeutic effectiveness of serious games since the results from this type of methods are intuitive and easy to analyze. Also, this type of method is more humane, more focusing on patients’ feelings. There are a sequence of standard quizzes, such as Mini-Mental State Examination (MMSE), Montreal Cognitive Assessment (MoCA), Confusion Assessment Method (CAM), Richmond Agitation-Sedation Scale (RASS), etc. Taking some references as examples, [54]–[57] used some standard questionnaires (e.g. MoCA, MMSE, CAM) or their self-created questionnaires to meet their different experimental requirements.

It is an objective, accurate and real-time method to test the effectiveness of the games through the patient’s physiological signals, because the reaction reflected by physiological signals is uncontrollable and close to the actual feelings of humans. Also, using real-time data to analyze patient’s status is more efficient. Some researchers used physiological signals to explore the effects of games on people. Wen *et al.* [16] and Qi *et al.* [17] collected EEG (electroencephalogram) signals to evaluate the condition of dementia patients. And Perugia *et al.* [58] collected EDA (electrodermal activity) of dementia patients during their interaction with social robots to explore the psychological response of them.

Moreover, we have added two components to this model, game results and professional review, to make the presented assessment model more professional and comprehensive. For one thing, observing and recording the game results of the elderly is a simple and straightforward way to assess the therapeutic effectiveness of games. We can keep our eyes on the changes in patients’ performance during the game to assess the therapeutic effectiveness of a certain game. For example, some game scores reflect players’ reaction ability; The accuracy of games represents the memory condition, problem solving ability and so on. For another, with a better understanding of the medical knowledge of dementia and the condition of their own patients, professionals can give better advice. So, it is essential that professionals join in the assessment of SGDC. For instance, a standard professional rating scale could be formulated to evaluate the performance of the elderly during the game in the future.

As described above, our model consists of four components, physiological signals, professional reviews, questionnaire tests, and game results. In our model, the assessment result will be obtained through the comprehensive assessment of these four modules. At the beginning of this assessment procedure, our goal is to collect data which can provide evidence for assessment. In this stage, we need to collect patients’ physiological signals during gaming. Additionally, professionals will be involved in this procedure to observe patients’ performance during the game and score each performance of patients (e.g. expression, action, etc.). Also, the game results of each game for every patient should be recorded by hand or game systems. When games are over, patients are required to finish some questionnaire tests (e.g. MMSE, MoCA, etc.). In the second stage, the processing and analysis of data are major tasks. For physiological signals, preprocessing, features extraction, features selection, and classification will be conducted [59], [60]. For the data from questionnaire tests, scores of performance and game results, statistics are applied in their analysis intensively. For instance, hypothesis testing can be used to analyze these data [61], [62]. In the final phase, the assessment result will be obtained by combining these analysis results of the above four components. However, there is no optimal weight for each component. In the future, we will conduct experiments to optimize this model and find the optimal weight for each component.

Especially, our model is designed to assess the therapeutic effectiveness of a certain game in its small-scale trial experiment. For the assessment of a game, our model needs to be performed twice to get two assessment results (the assessment result before the trial and the assessment result after a period of trial). Then, these two assessment results will be compared by independent t-test. If the result of the independent t-test shows that there is a significant difference, the game can be put into use widely and formally. In other words, whether the results of the independent t-test are significantly different is the benchmark for judging whether the game is effective.

Compared with other assessment methods, the main difference of our model is multi-group participation and multi-method combination which is also the distinct advantage of our model. For example, [63], [64] applied questionnaire tests into assessments in which only the patients’ participation is involved; [65], [66] used physiological signals to analyze the conditions of patients which only refers to an assessment method, physiological signals analysis. These above assessments all involved one group participation or one method application which may make the assessment result accidental, whereas our model contains multi-group participation and multi-method combination. Theoretically, this kind of assessment with multi-component combination allows each component to correct and supplement each other which enables our model more comprehensive, professional and reliable.

## IV. Discussion and Prospect

Serious games have an important place in dementia care and plenty of related studies are presented. Various kinds of games can be applied into dementia care, namely board games, video games, virtual reality games. Also, a broad range of techniques are used to develop games, among which are Somatosensory technology, 3D graphics generation technology, sensor technology and so on. However, serious games are no panacea for dementia care. Since they are more effective in treating early-stage and middle-stage dementia while more futile faced with late-stage dementia. Likewise, the research on SGDC mainly focuses on the treatment of early-stage or middle-stage dementia. Besides, although there are abundant research results, a unified category and the standard assessment model of therapeutic effectiveness both are not available for SGDC. Considering these challenges, the following directions are worth to be further developed.

• More therapies, like music therapy, reminiscence therapy, can be integrated with serious games to improve effectiveness of games therapy and provide more new ideas for the design of serious games.
• A Serious Games Hospital for dementia care can be established to offer systematic and professional treatment for patients with dementia. For instance, different departments can be set according to different symptoms in Serious Game Hospital.
• To give an explicit guideline for researchers, clear category architecture should be presented. For example, the category can be based on different symptoms, such as mild, moderate or severe cognitive impairments along with some integrated symptoms.
• To choose effective serious games for patients, the professional assessment model deserves further study. Researchers can pay more attention to multi-group participation (e.g. patients, professionals, etc.) and multi-method combination (e.g. questionnaire tests, physiological signals, etc.).
• For some common video games, patients can play on their own at home. Whereas, for some VR games with expensive equipment, most patients can not play at home by themselves. In the face of this situation, the hospital can equip the community with relevant resources, which not only allows more patients to obtain abundant medical resources but also reduces the burden on the hospital. Moreover, this also requires the government to make relevant public interest policies to support.
• SGDC is a cross-field of computer science and medical science. In the development of SGDC, game designers and medical researchers need to maintain frequent and close communication, which can design interesting and effective serious games. In addition, the cultivation of talents must be promoted at the intersections of computer science and medical science.

## V. Conclusion

As an effective treatment for dementia, SGDC is intensively studied. However, with the long-term study, there is no systematic review of the development stage for SGDC. To fill this gap, we investigate the development route of SGDC and divided its development into 3 stages: board games stage, video games stage and VR games stage, which can help researchers to better understand its development quickly. Apart from that, we found that a unified category framework of SGDC is still unavailable to regulate its development. Considering this, we analyze characteristics of dementia symptoms of different stages and present a category of SGDC based on it to encourage developers to design more symptom-targeted serious games. What’s more, it is essential for an effective treatment that reliable assessment methods should be formulated. Given that, we review existing assessment methods and present an assessment model with multi-group participation and multi-method combination which is more comprehensive than those existing methods. Finally, we discuss the present SGDC and its challenges and put forward 6 directions worthy of further investigation. In conclusion, our work aims at helping researchers to have a better understanding of SDGC’s development and to design more effective serious games for dementia care.
